# Editorial: 2D Materials for Advanced Sensors: Fabrication and Applications

**DOI:** 10.3390/nano15030180

**Published:** 2025-01-23

**Authors:** Wugang Liao, Lin Wang

**Affiliations:** 1 State Key Laboratory of Radio Frequency Heterogeneous Integration, College of Electronics and Information Engineering, Shenzhen University, Shenzhen 518060, China; 2 School of Mechanical Engineering, Shanghai Jiao Tong University, Shanghai 200240, China

The rapid advancements in the field of two-dimensional (2D) materials have significantly influenced the development of innovative sensor technologies. Since the isolation of graphene, 2D materials have attracted considerable attention due to their remarkable properties—atomic thinness, exceptional mechanical strength, high surface-to-volume ratios, and tunable electronic characteristics—making them ideal candidates for next-generation sensors. This makes them particularly suitable for applications in chemical sensing, gas detection, thermal and pressure sensing, photodetection, flexible sensors, etc. These unique attributes position 2D materials as key components for sensors used in the rapidly expanding Internet of Things (IoT) ecosystem.

**Fabrication of 2D Materials**

The development of advanced fabrication methods for 2D materials is crucial for their integration into real-world sensor devices. Several contributions in this issue focus on novel approaches to the synthesis and processing of 2D materials, highlighting significant progress in overcoming challenges related to scalable production and functionalization.

Korotcenkov et al. [1] provide a thorough overview of porous 2D nanomaterials, particularly for gas sensing applications. Their work underscores the importance of nanostructuring in enhancing sensor performance, particularly in terms of response time and sensitivity. While these materials show great promise, the authors also identify challenges in applying them to practical sensor devices, highlighting the need for continued innovation in fabrication techniques. Liu et al. [2] review hybrid 1D/2D nanostructures, which demonstrate superior performance in sensors for various applications, including gas detection, photodetection, and biosensing. Their review provides an in-depth analysis of the fabrication of these hybrid materials and their application potential, addressing both the challenges and vast opportunities in this field.

In addition, an important contribution to the fabrication of 2D materials is made by Novikova et al. [3], who explore a sustainable and green extraction method for producing high-quality graphene from natural shungite. This environmentally friendly approach presents a scalable, low-cost alternative to traditional graphene production methods, making it an attractive option for the large-scale manufacturing of 2D materials for sensor applications. Furthermore, Kim et al. [4] present a novel direct chemical vapor deposition (CVD) method for the synthesis of transferable 3D graphene using a transfer-support layer (TSL) on sapphire substrate (PSS). This method overcomes the limitations of traditional wet-transfer techniques, which often result in structural damage to graphene. By utilizing the TSL as a stabilizing agent, they achieve the growth of large-area, high-quality 3D graphene that can be easily transferred onto flexible substrates. This efficient process preserves the mechanical integrity of the 3D graphene structure, holding great promise for the development of flexible and wearable sensor technologies.

**Applications of 2D Materials in Advanced Sensors**

The unique properties of 2D materials make them ideal candidates for a wide range of sensor applications. Their ultra-thin nature, high surface-to-volume ratios, and tunable electronic properties enable them to be highly sensitive to external stimuli, making them well suited for sensing applications across various fields, including chemical sensing, gas detection, environmental monitoring, and healthcare.

Graphene-based sensors are particularly attractive because of their high sensitivity and rapid response time. For example, Khaleghiabbasabadi et al. [5] develop a high-performance NO_2_ sensor using a reduced graphene oxide-based composite enhanced by Fe_3_O_4_ and piperidine-4-sulfonic acid. Their work emphasizes the importance of material selection in achieving superior sensitivity, particularly for air quality monitoring, where the precise detection of NO_2_ is essential for environmental health. Furthermore, Kim et al. [4] also make a significant contribution by demonstrating the application of 3D graphene in flexible surface-enhanced Raman spectroscopy (SERS) sensors. They utilize a novel CVD-based fabrication method to integrate 3D graphene onto flexible substrates, resulting in highly sensitive and reusable SERS sensors. The ability to reuse these sensors without compromising performance is a major advantage for portable, flexible, and cost-effective sensing devices, making them suitable for healthcare and environmental applications where high sensitivity and reusability are necessary.

Another important contribution comes from Novikova et al. [6], who investigate the protective capabilities of graphene for optical microfibers. By applying a graphene film mixed with gold nanoparticles, they significantly enhance the durability and operational lifetime of optical sensors. This development is crucial for improving the long-term stability and performance of sensors used in real-time applications, such as optical communication and environmental sensing, where sensor longevity and reliability are critical. In addition, Zhang et al. [7] demonstrate the potential of carbon nanotube (CNT)-based micropolarizer arrays (MPAs), which use highly aligned CNT films to enable real-time polarization measurements. This work exemplifies how carbon-based materials can be integrated into optical sensing technologies, particularly in biosensing and imaging applications, where polarization plays a key role in enhancing signal quality.

Transition metal dichalcogenides (TMDCs), such as MoS_2_, have also emerged as promising materials for sensor applications. These 2D materials offer distinct advantages due to their tunable electronic and optical properties, making them ideal for applications in gas detection and photodetection. For example, Zhou et al. [8] present a MoS_2_/graphene van der Waals heterojunction-based surface plasmon resonance (SPR) sensor, which demonstrates enhanced sensitivity for gas molecule detection. The combination of MoS_2_ and graphene significantly improves the electric field enhancement at the sensing interface, leading to a more efficient and sensitive platform for environmental monitoring and chemical sensing. Hong et al. [9] develop MoS_2_-based sensors for respiration monitoring in healthcare applications. Their study demonstrates how thermal annealing can significantly improve the sensor’s response time, enhancing its accuracy and reliability for portable medical diagnostics. MoS_2_ and other TMDCs have also shown considerable promise in the field of versatile heterostructure photodetection. Huang et al. [10] explore the use of MoS_2_/PdSe_2_ heterostructures for developing a reconfigurable polarimetric photodetector. Their work demonstrates the ability to achieve tunable responsivity and polarization control, which could greatly enhance imaging systems and photodetection applications.

In addition to graphene and TMDCs, other 2D materials, such as borophene, are gaining attention for their potential in sensor applications. Duan et al. [11] demonstrate the gas-sensing capabilities of borophene, showing, through density functional theory (DFT) calculations, that borophene exhibits exceptional sensitivity to gases like CO, NH_3_, SO_2_, H_2_S, and NO_2_. This creates exciting possibilities for environmental monitoring, where sensitive and selective gas detection is essential. Furthermore, Ermolaev et al. [12] investigate the optical properties of van der Waals materials, particularly Bi_2_Se_3_, which exhibit consistent optical constants across various synthesis methods. Their findings are crucial for photonic applications, such as biosensing and nanoparticle therapy, where the reproducibility and reliability of optical properties are essential for reliable performance in real-world settings.

In summary, the Special Issue *“2D Materials for Advanced Sensors: Fabrication and Applications”* reflects the rapid developments and innovations in the fabrication, characterization, and application of 2D materials in sensor technologies. This issue not only highlights significant advancements in the development of new fabrication methods—such as green extraction techniques and advanced CVD processes—but also underscores the diverse applications of these materials across various sensing domains, including chemical, gas, thermal, and flexible sensors.

The findings presented in this Special Issue are expected to stimulate further research in the field of 2D materials and their integration into advanced sensor systems. They provide a multifaceted perspective on the potential of these materials to revolutionize sensing technologies for the Internet of Things (IoT), environmental monitoring, healthcare, and beyond. The contributions showcased here illustrate the vast range of possibilities for 2D material-based sensors, paving the way for more sustainable, flexible, and highly sensitive devices.
